# A Novel Role for Bcl-2 in Regulation of Cellular Calcium Extrusion

**DOI:** 10.1016/j.cub.2012.05.002

**Published:** 2012-07-10

**Authors:** Pawel E. Ferdek, Julia V. Gerasimenko, Shuang Peng, Alexei V. Tepikin, Ole H. Petersen, Oleg V. Gerasimenko

**Affiliations:** 1 Medical Research Council Group, School of Biosciences, Cardiff University, Cardiff CF10 3AX, Wales, UK; 2 Department of Physiology, Medical College, Jinan University, Guangzhou 510632, China; 3 The Physiological Laboratory, Department of Cellular and Molecular Physiology, Institute of Translational Medicine, University of Liverpool, Liverpool L69 3BX, UK

## Abstract

The antiapoptotic protein Bcl-2 [1, 2] plays important roles in Ca^2+^ signaling [3] by influencing inositol triphosphate receptors and regulating Ca^2+^-induced Ca^2+^ release [4–6]. Here we investigated whether Bcl-2 affects Ca^2+^ extrusion in pancreatic acinar cells. We specifically blocked the Ca^2+^ pumps in the endoplasmic reticulum and assessed the rate at which the cells reduced an elevated cytosolic Ca^2+^ concentration after a period of enhanced Ca^2+^ entry. Because external Ca^2+^ was removed and endoplasmic reticulum Ca^2+^ pumps were blocked, Ca^2+^ extrusion was the only process responsible for recovery. Cells lacking Bcl-2 restored the basal cytosolic Ca^2+^ level much faster than control cells. The enhanced Ca^2+^ extrusion in cells from Bcl-2 knockout (Bcl-2 KO) mice was not due to increased Na^+^/Ca^2+^ exchange activity, because removal of external Na^+^ did not influence the Ca^2+^ extrusion rate. Overexpression of Bcl-2 in the pancreatic acinar cell line AR42J decreased Ca^2+^ extrusion, whereas silencing Bcl-2 expression (siRNA) had the opposite effect. Loss of Bcl-2, while increasing Ca^2+^ extrusion, dramatically decreased necrosis and promoted apoptosis induced by oxidative stress, whereas specific inhibition of Ca^2+^ pumps in the plasma membrane (PMCA) with caloxin 3A1 reduced Ca^2+^ extrusion and increased necrosis. Bcl-2 regulates PMCA function in pancreatic acinar cells and thereby influences cell fate.

## Highlights

► Bcl-2 reduces Ca^2+^ extrusion through PMCA in control cells as compared to Bcl-2 KO ► Bcl-2 reduces Ca^2+^ extrusion though PMCA in AR42J cells overexpressing Bcl-2 ► Loss of Bcl-2 reduces Ca^2+^ overload, decreases necrosis, and promotes apoptosis

## Results and Discussion

### Loss of Bcl-2 Affects Ca^2+^ Signaling in Pancreatic Acinar Cells

**Figure 1:**
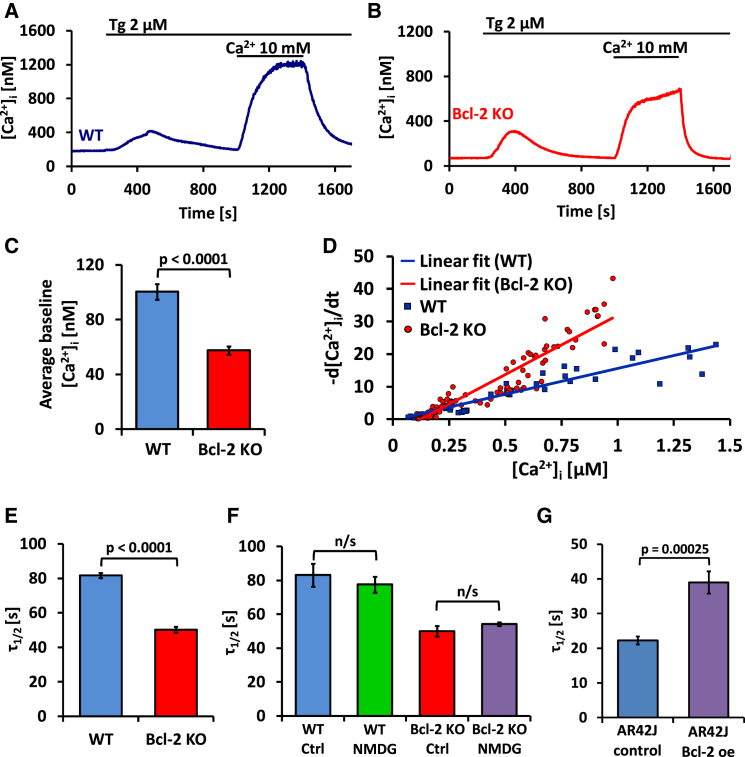
Loss of Bcl-2 Protein Is Associated with Increased Na^+^-Independent Ca^2+^ Extrusion across the Plasma Membrane (A) Typical [Ca^2+^]_i_ trace recorded in a normal (WT) pancreatic acinar cell. Changes in [Ca^2+^]_i_ were evoked first by application of thapsigargin (Tg) in the absence of external Ca^2+^ and thereafter by exposure, for a period of 400 s, to an external solution containing 10 mM Ca^2+^. The reduction in the elevated [Ca^2+^]_i_ following removal of the high Ca^2+^ external solution can, in the continued presence of Tg, only be due to Ca^2+^ extrusion across the plasma membrane. (B) Pancreatic acinar cell from Bcl-2 KO mouse. The same protocol was used as in (A). The rate of reducing [Ca^2+^]_i_ (due to Ca^2+^ extrusion) after removal of 10 mM external Ca^2+^ was much faster than in the WT cell (shown in A). The resting [Ca^2+^]_i_ was also lower than in the WT cell. (C) Comparison of the initial (resting, baseline) [Ca^2+^]_i_ in WT (blue bar, n = 34) and Bcl-2 KO (red bar, n = 109) pancreatic acinar cells (p < 0.0001). Data in (C) and (E)–(G) are presented as mean ± SEM. (D) Dependence of the initial rate of Ca^2+^ extrusion on [Ca^2+^]_i_, calculated from experiments of the type shown in (A) and (B), i.e., WT (blue, n = 34) and Bcl-2 KO (red, n = 109) pancreatic acinar cells. In cells from Bcl-2 KO mice, Ca^2+^ extrusion was much faster. See also Figure S1. (E) Bar chart comparing half-times (τ_1/2_) of the reduction in [Ca^2+^]_i_ toward the resting level following removal of external Ca^2+^ in WT (blue bar, n = 20) and Bcl-2 KO (red bar, n = 38) cells. (F) Bar chart comparing half-times (τ_1/2_) of the reduction in [Ca^2+^]_i_ toward the resting level following removal of external Ca^2+^ in WT pancreatic acinar cells in the normal presence of external Na^+^ (blue bar, n = 18) with those obtained when external Na^+^ was replaced by NMDG^+^ (green bar, n = 24) as well as in Bcl-2 KO cells (red bar, in the presence of Na^+^, n = 6; purple bar, when Na^+^ was replaced by NMDG^+^, n = 25). See also Figure S2. (G) Bar chart comparing the half-times (τ_1/2_) of the reduction in [Ca^2+^]_i_ toward the resting level following removal of external Ca^2+^ in control AR42J cells (blue bar, n = 14) and in AR42J cells transfected with pcDNA3 Bcl-2 plasmid (Bcl-2 overexpression [oe]) (purple bar, n = 17). Data were collected from cells expressing cytosolic Cameleon YC3.60. See also Figure S3.

In this study, we compared Ca^2+^ signaling mechanisms, with a particular emphasis on Ca^2+^ extrusion, in pancreatic acinar cells isolated from Bcl-2 knockout (Bcl-2 KO) and control mice. We used a protocol allowing us to monitor the rate of reducing the cytosolic Ca^2+^ concentration ([Ca^2+^]_i_) following a maximal elevation of [Ca^2+^]_i_ during complete blockade of Ca^2+^ uptake into the endoplasmic reticulum (ER) (Figures 1A and 1B). Wild-type (WT) and Bcl-2 KO cells exposed to a Ca^2+^-free solution were treated with thapsigargin (Tg), a specific inhibitor of Ca^2+^ pumps in the ER, in order to empty the ER Ca^2+^ stores. The extracellular Ca^2+^ concentration ([Ca^2+^]_o_) was then increased to 1 mM, 5 mM, or 10 mM, which induced rapid influx of Ca^2+^ to the cytosol. After a stable [Ca^2+^]_i_ plateau had been attained, extracellular Ca^2+^ was removed and [Ca^2+^]_i_ declined until the baseline level had been reestablished (Figures 1A and 1B). Comparing the initial [Ca^2+^]_i_ of WT and Bcl-2 KO cells, as shown in Figures 1A and 1B, we found that Bcl-2 KO cells had a significantly lower [Ca^2+^]_i_ (57.5 ± 3 nM SE) than WT cells (100.3 ± 5.6 nM SE) (Figure 1C). This significant difference suggests important changes in equilibrium between Ca^2+^ entry and extrusion across the plasma membrane.

### Loss of Bcl-2 Enhances Ca^2+^ Extrusion across the Plasma Membrane

Because Ca^2+^ uptake into the ER was blocked by Tg, the rate of decline of [Ca^2+^]_i_—after removal of external Ca^2+^—must reflect the rate of Ca^2+^ extrusion. Calcium extrusion rates (d[Ca^2+^]_i_/dt) were calculated and then plotted as a function of the [Ca^2+^]_i_ values obtained from the exponential fit. Initial d[Ca^2+^]_i_/dt values (for the highest [Ca^2+^]_i_) from each trace were plotted together on a graph and fitted by linear regression (Figure 1D). Similar results were obtained when we compared rates of recovery in cells treated with a high dose of Tg (10 μM) and acetylcholine (ACh) (see Figures S1A and S1B available online) or Tg alone (Figures S1C and S1D). The half-time of recovery of baseline [Ca^2+^]_i_ levels in the experiments shown in Figures 1A and 1B was calculated, and the results demonstrate that recovery in Bcl-2 KO cells was much faster than in WT cells (Figure 1E).

### Ca^2+^ Extrusion in Pancreatic Acinar Cells Is Mainly Dependent on Plasma Membrane Calcium-Activated ATPase

Tg blocks very specifically the ER Ca^2+^ pumps (sarcoendoplasmic reticulum Ca^2+^-activated ATPase [SERCA]). In such conditions, Ca^2+^ removal from the cytosol is only dependent on Ca^2+^ export across the plasma membrane. The two main proteins responsible for this process are the plasma membrane calcium-activated ATPase (PMCA) [7] and the Na^+^/Ca^2+^ exchanger (NCX) [8]. However, available evidence indicates that NCX plays a very minor, if any, role in Ca^2+^ extrusion from normal pancreatic acinar cells [9] and that therefore Ca^2+^ removal across the plasma membrane in these cells is mediated by PMCA.

### Na^+^/Ca^2+^ Exchange Does Not Contribute to Ca^2+^ Extrusion in WT and Bcl-2 KO Cells

In order to test independently, in pancreatic acinar cells, whether NCX plays any significant role in Ca^2+^ extrusion, we performed two different series of experiments. In the first, we replaced all Na^+^ in the external solution with N-methyl D-glucamine (NMDG^+^). This approach has been used frequently to inhibit NCX in the plasma membrane [10, 11]. We compared the Ca^2+^ extrusion rates, following removal of external Ca^2+^ in the presence of Tg, in WT (Figures S2A and S2B) and Bcl-2 KO cells (Figures S2C and S2D) and summarized the results by calculating the average half-time of recovery of the basal [Ca^2+^]_i_ (Figure 1F). In experiments on both WT and Bcl-2 KO cells, removal of external Na^+^ had no effect on the rate of Ca^2+^ extrusion, as the half-times of the recovery of the basal [Ca^2+^]_i_ were very similar to controls when Na^+^ was replaced by NMDG^+^. Ca^2+^ extrusion across the plasma membrane is therefore independent of the presence or absence of external Na^+^, and the enhanced rate of Ca^2+^ extrusion observed in Bcl-2 KO cells cannot be explained by recruitment of NCX but must be due to increased Ca^2+^ outflux mediated by PMCA.

In the second series of experiments, we used LaCl_3_ as a blocker of PMCA. It has previously been shown that 1 mM lanthanum abolishes Ca^2+^ extrusion by PMCA without affecting NCX [12]. As seen in Figures S2E and S2F, there was no decline of [Ca^2+^]_i_ following removal of external Ca^2+^ in the presence of La^3+^ but, as soon as the trivalent cation had been removed, [Ca^2+^]_i_ started to decrease. These experiments confirm that NCX has at most a very minor role in cytosolic Ca^2+^ clearance in pancreatic acinar cells.

### Overexpression of Bcl-2 Slows Down Ca^2+^ Extrusion in AR42J Cells

**Figure 2:**
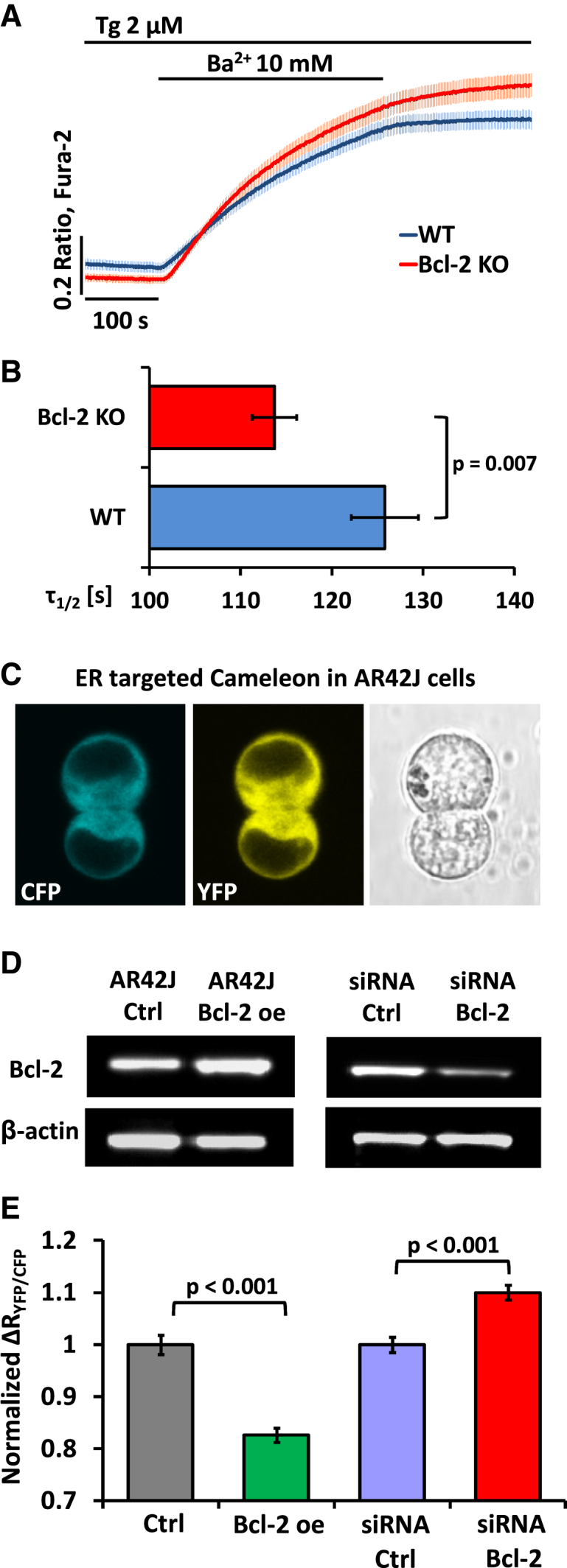
Effects of Bcl-2 on Ca^2+^/Ba^2+^ Influx and [Ca^2+^]_ER_ (A) Changes in the average [Ba^2+^]_i_ following introduction of an external solution containing 10 mM Ba^2+^ in the continued presence of Tg, recorded in WT (blue trace, n = 60) and Bcl-2 KO (red trace, n = 61) pancreatic acinar cells. Traces are shown with standard errors. Data in (A), (B), and (E) are presented as mean ± SEM. (B) Bar chart comparing half-times (τ_1/2_) of the rise in Ba^2+^-induced Fura-2 ratio toward a plateau in WT (blue bar, n = 60) and Bcl-2 KO (red bar, n = 61) pancreatic acinar cells (shown in A). (C) An AR42J cell doublet transfected with D1ER Cameleon. Blue fluorescence comes from the CFP component and yellow fluorescence from YFP. (D) Results of immunoblotting against Bcl-2 performed on total protein isolated from (from left to right) control untransfected AR42J cells, AR42J cells stably transfected with pcDNA3 plasmid containing human Bcl-2 insert, AR42J cells transfected with control (scrambled) siRNA, and AR42J cells transfected with Bcl-2 siRNA. β-actin is shown as loading control. See also Figure S4 for distribution of Bcl-2 in pancreatic acinar cells and AR42J cells. (E) Bar chart comparing differences in resting [Ca^2+^]_ER_, presented as the difference between basal YFP/CFP values and YFP/CFP values obtained after treatment with 20 μM CPA, normalized to controls. The gray bar represents control, untransfected AR42J cells (n = 78), the green bar represents cells transfected with pcDNA3 plasmid containing human Bcl-2 insert (n = 97), the blue bar represents AR42J cells transfected with scrambled siRNA (n = 69), and the red bar represents AR42J cells transfected with Bcl-2 siRNA (n = 62).

In order to test further the role of Bcl-2 in regulating Ca^2+^ extrusion, we stably overexpressed Bcl-2 in a pancreatic acinar cell line (AR42J) and then assessed the rate of recovering the basal [Ca^2+^]_i_ after an imposed period of elevated [Ca^2+^]_i_ using our standard protocol (Figures 1A and 1B). In these experiments we measured [Ca^2+^]_i_ using two independent methods: cytoplasmic calcium Cameleon YC3.60 (Figures 1G, S3A, and S3B) and Fura-2 (Figures S3C–S3E). Ca^2+^ extrusion in cells overexpressing Bcl-2 was substantially slower than in control cells in both these series of experiments (Figures 1G and S3E), confirming a role for Bcl-2 in regulation of Ca^2+^ extrusion. Bcl-2 must act by inhibiting PMCA because in these cells, Ca^2+^ extrusion was also independent of the presence or absence of external Na^+^ (Figures S3A–S3D). The increased level of Bcl-2 in the overexpressing cells was confirmed by western blot analysis (Figure 2D).

### Bcl-2 Regulates Ca^2+^/Ba^2+^ Influx in Pancreatic Acinar Cells

In the presence of external Ca^2+^, there will inevitably be both Ca^2+^ entry and Ca^2+^ extrusion, and it was therefore of interest also to test the possible effect of Bcl-2 on Ca^2+^ entry alone. Because Ba^2+^ is not extruded by the Ca^2+^ pump but does pass through Ca^2+^ channels, we used Ba^2+^ in the external solution rather than Ca^2+^ in these experiments [13]. Comparisons of the averaged Ba^2+^ entry traces (Figure 2A) and the half-times of the rises in the intracellular [Ba^2+^] (Figure 2B) show that the rate of Ba^2+^ entry is significantly enhanced in Bcl-2 KO cells as compared to WT cells. However, the effect of Bcl-2 on Ca^2+^/Ba^2+^ entry is quantitatively much smaller than the effect on Ca^2+^ extrusion (Figures 1A, 1B, and 1E).

### Bcl-2 Regulates the Resting Ca^2+^ Levels in the ER

It was shown previously [3, 14] that Bcl-2 overexpression reduces Ca^2+^ loading of the ER stores affecting Ca^2+^ leak and uptake. This has been confirmed by some laboratories [15, 16], but not by others [17, 18]. We measured the resting [Ca^2+^] in the ER ([Ca^2+^]_ER_) of AR42J cells [19] with ER-targeted D1ER Cameleon (Figure 2C) [16, 20, 21] in untransfected AR42J cells, cells stably transfected with human Bcl-2, cells transiently transfected with Bcl-2-siRNA, and cells transiently transfected with control (scrambled) siRNA. The relative levels of Bcl-2 in control and Bcl-2-overexpressing AR42J cells are shown in Figure 2D. Cells from each group were treated with the SERCA inhibitor cyclopiazonic acid (CPA) in order to liberate the releasable Ca^2+^ from the ER, and changes in the ratio YFP/CFP were compared. The results shown in Figure 2E demonstrate that Bcl-2 overexpression caused a decrease in resting [Ca^2+^]_ER_ (0.83 ± 0.013 SE, n = 97, p < 0.001 as compared to 1.00 ± 0.018 SE, n = 78 in untransfected control cells; arbitrary units), whereas the opposite effect was obtained by knocking down Bcl-2 with specific siRNA (1.10 ± 0.014 SE, n = 62, p < 0.001 as compared to 1.00 ± 0.015 SE, n = 69 with scrambled siRNA; arbitrary units). Our data are in line with the majority of previous studies [3, 14–16] suggesting that the Ca^2+^ leak channel in the ER [3, 15] and possibly the SERCA [22, 23] are regulated by Bcl-2. Our finding also underlines the necessity to assess [Ca^2+^]_ER_ directly, because indirect cytosolic measurements can be misleading with regard to information about ER Ca^2+^ loading.

### Loss of Bcl-2 Protects against Necrosis and Promotes Apoptosis

**Figure 3:**
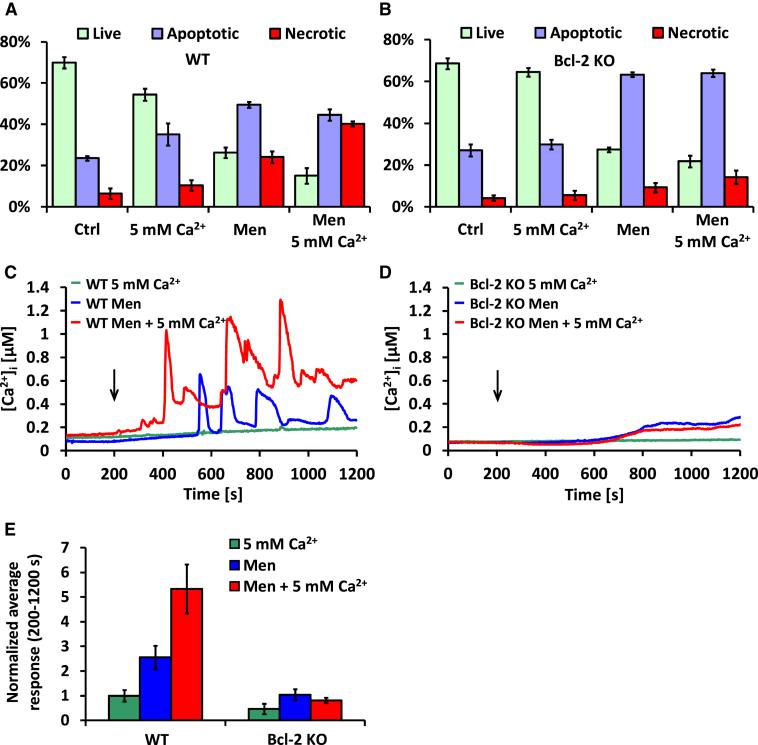
Bcl-2-Induced Inhibition of Ca^2+^ Extrusion Is Responsible for Higher Levels of Necrosis When Cells Are Stressed by Raised External [Ca^2+^] and/or by Menadione (A) Results of cell death assays performed on WT pancreatic acinar cells. Light green bars represent live cells, blue bars represent apoptotic cells, and red bars represent necrotic cells. The chart is separated into four treatment groups, from the left: untreated control cells, cells treated with 5 mM Ca^2+^, cells treated with 30 μM menadione, and cells treated with both 30 μM menadione and 5 mM Ca^2+^. Error bars in (A), (B), and (E) represent SEM. (B) Results of cell death assays performed on Bcl-2 KO pancreatic acinar cells. The chart is structured in the same way as in (A), which allows comparison of the differences between WT and Bcl-2 KO cells. Cells lacking Bcl-2 undergo more apoptosis and less necrosis as compared to WT cells when challenged with 30 μM menadione or both menadione and high Ca^2+^. (C) Typical [Ca^2+^]_i_ responses to 5 mM Ca^2+^ (green, n_WT_ = 18), 30 μM menadione in 1 mM Ca^2+^ solution (blue, n_WT_ = 23), and 30 μM menadione in 5 mM Ca^2+^ (red, n_WT_ = 22) in WT pancreatic acinar cells. Black arrow shows time point when treatment was applied. (D) Typical [Ca^2+^]_i_ responses to 5 mM Ca^2+^ (green, n_Bcl-2_ = 12), 30 μM menadione in 1 mM Ca^2+^ solution (blue, n_Bcl-2_ = 14), and 30 μM menadione in 5 mM Ca^2+^ (red, n_Bcl-2_ = 12) in Bcl-2 KO pancreatic acinar cells. Black arrow shows time point when treatment was applied. (E) The responses depicted in (C) and (D) were quantitatively analyzed and shown as the average [Ca^2+^]_i_ responses above the baseline recorded between 200 and 1,200 s and then normalized to the average value of responses to 5 mM Ca^2+^ in WT cells. Green bars represent cells treated with 5 mM Ca^2+^, blue bars represent cells treated with 30 μM menadione, and red bars represent cells treated with both 30 μM menadione and 5 mM Ca^2+^. Responses of WT cells to menadione were greater than those recorded in Bcl-2 KO cells and were additionally potentiated by high extracellular Ca^2+^.

In order to investigate the pathophysiological importance of Bcl-2 and in particular its regulation of Ca^2+^ extrusion, we performed cell death assays on freshly isolated WT and Bcl-2 KO pancreatic acinar cells treated with 30 μM menadione [24] and/or high external [Ca^2+^] (5 mM) for 30 min. Control groups were incubated for 30 min without any treatment. Our results (Figures 3A and 3B) show that untreated WT and Bcl-2 KO cells are characterized by very similar levels of apoptosis and necrosis. A high (5 mM) external [Ca^2+^] reduced the number of live WT cells in comparison to WT controls (p = 0.019). The same phenomenon was not observed in Bcl-2 KO cells (p = 0.05). Menadione (30 μM) caused a substantial reduction in live WT cells in comparison to WT controls (p < 0.001), increasing both apoptosis (p < 0.001) and necrosis (p = 0.01). Treatment of WT cells with both menadione and high Ca^2+^ increased necrosis even more than treatment with menadione alone (p = 0.018). Bcl-2 KO cells treated with 30 μM menadione had decreased numbers of live cells (p = 0.001) and markedly increased numbers of apoptotic cells (p = 0.003) in comparison to the untreated Bcl-2 KO cells. However, necrosis levels were not significantly different when compared to the Bcl-2 KO control (p = 0.138). Combined treatment with menadione and 5 mM Ca^2+^ did not substantially increase necrosis levels above those induced by menadione alone (p = 0.282). Importantly, Bcl-2 KO cells treated with menadione had increased apoptosis levels and decreased necrosis levels as compared to WT cells treated in the same way (p = 0.002 and p = 0.016, respectively). Necrosis inhibition in Bcl-2 KO versus WT cells was even more pronounced between the groups incubated with menadione and high Ca^2+^ (p = 0.008).

We also compared the rise in [Ca^2+^]_i_ in Fura-2-loaded WT cells and Bcl-2 KO cells in response to menadione (30 μM) or to raising the external [Ca^2+^] from 1 to 5 mM or to both raising [Ca^2+^]_o_ to 5 mM and simultaneously adding menadione. Typical traces are shown in Figures 3C and 3D. An increase in [Ca^2+^]_o_ from 1 mM to 5 mM resulted in a small, slowly developing elevation of [Ca^2+^]_i_ in WT cells but had almost no effect on Bcl-2 KO cells (green traces, Figures 3C and 3D). In both WT and Bcl-2 KO cells, treatment with menadione caused a slow elevation of [Ca^2+^]_i_. However, WT cells additionally responded with robust oscillations on top of the increasing [Ca^2+^]_i_ baseline (blue trace, Figure 3C). These oscillations were markedly potentiated in the presence of 5 mM Ca^2+^ (red trace, Figure 3C). In contrast, cells lacking functional Bcl-2 protein did not develop such oscillations in response to menadione (blue trace, Figure 3D), and an increased [Ca^2+^]_o_ had no further effect on menadione-elicited responses in those cells (red trace, Figure 3D). Figure 3E summarizes the effects of menadione and high extracellular Ca^2+^ on WT and Bcl-2 KO cells. The responses are presented as averaged areas under the traces between 200 and 1,200 s calculated in the same way for each treatment and subsequently normalized to the average values of responses to 5 mM Ca^2+^ in WT cells. Responses to 5 mM Ca^2+^ appear to be lower in Bcl-2 KO cells as compared to WT cells, although the difference is not statistically significant (p = 0.105). Menadione applied in the presence of both 1 mM and 5 mM extracellular Ca^2+^ induced much larger responses in WT cells as compared to Bcl-2 KO cells (p = 0.007 and p < 0.001, respectively). In WT cells, the presence of 5 mM Ca^2+^ together with menadione caused much larger responses than menadione alone (p = 0.017), whereas in Bcl-2 KO cells, the presence of 5 mM Ca^2+^ did not affect menadione-dependent responses (p = 0.38). These data demonstrate that removal of Bcl-2 affords a remarkable degree of protection against attempts to raise [Ca^2+^]_i_ by elevation of the external Ca^2+^ and/or stimulation with menadione.

### Inhibition of PMCA by Caloxin 3A1 Promotes Necrosis

**Figure 4:**
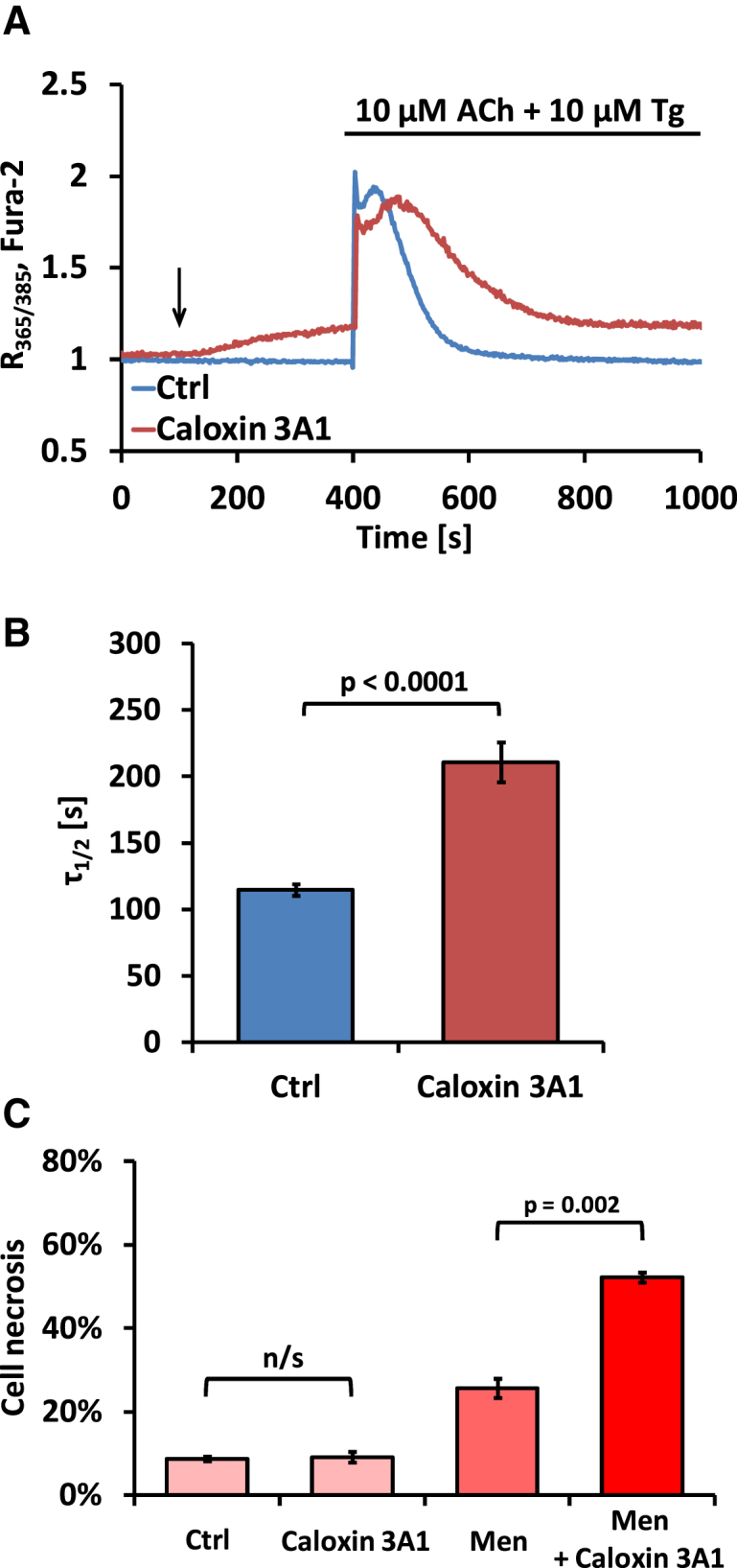
Specific Inhibition of PMCA Substantially Increases Necrosis in Pancreatic Acinar Cells (A) Typical [Ca^2+^]_i_ changes in response to 10 μM Tg and 10 μM acetylcholine (ACh) in an untreated pancreatic WT pancreatic acinar cell (blue trace) or a WT cell exposed to 1 mM caloxin 3A1 (dark red trace). Black arrow indicates time of caloxin 3A1 application. Note the gradual increase in [Ca^2+^]_i_ after caloxin 3A1 application. (B) Bar chart comparing half-times (τ_1/2_) of the reduction in [Ca^2+^]_i_ to the resting level after responses to 10 μM Tg and 10 μM ACh in the presence (dark red bar, n = 26) or absence (blue bar, n = 15) of 1 mM caloxin 3A1. Error bars in (B) and (C) represent SEM. (C) Bar chart comparing necrosis levels in WT pancreatic acinar cells under different conditions. Cells were incubated for 30 min in the presence of 1 mM caloxin 3A1, 30 μM menadione, or 1 mM caloxin 3A1 and 30 μM menadione. Control cells were incubated for the same amount of time, but without any treatment.

We studied the physiological importance of PMCA-mediated Ca^2+^ extrusion by using the specific PMCA inhibitor peptide caloxin 3A1 [25]. Figure 4A shows typical changes in [Ca^2+^]_i_ in response to Tg and ACh in normal control pancreatic acinar cells as compared to cells preincubated for 300 s in the presence of caloxin 3A1. Caloxin 3A1 increased the basal resting [Ca^2+^]_i_ and significantly inhibited Ca^2+^ extrusion (Figure 4A), effectively doubling the half-time of recovery of the prestimulation [Ca^2+^]_i_ (Figure 4B). Caloxin 3A1 by itself did not increase the proportion of necrotic cells (Figure 4C) during relatively short (30 min) experiments, but it dramatically increased the proportion of necrotic cells when it was combined with menadione (Figure 4C).

### Localization of Bcl-2

The mechanism by which Bcl-2 influences PMCA-mediated Ca^2+^ extrusion from pancreatic acinar cells needs to be explored in future studies. Localization studies (Figures S4A–S4C) indicate that Bcl-2 is widely expressed, in both pancreatic acinar cells and AR42J cells, including in the ER. Given that it is clear that the ER can come very close to the plasma membrane in pancreatic acinar cells [26], it is not surprising that PMCA and Bcl-2 are partly colocalized (Figures S4A–S4C). This clearly cannot be taken as evidence for a direct interaction between these two proteins but does not exclude such an interaction.

### Conclusions

Our results reveal a new and unexpected role for Bcl-2 in the regulation of cellular Ca^2+^ homeostasis. In addition to the previously reported effects on Ca^2+^ signaling [3–5], we now show that Bcl-2 can suppress PMCA-mediated cellular Ca^2+^ extrusion and that such an inhibition has consequences for cell fate. Our data show that Bcl-2 KO cells are protected against the adverse effects of high extracellular Ca^2+^, because they can extrude cytosolic Ca^2+^ more efficiently than WT cells. Furthermore, loss of Bcl-2 protein strongly promotes apoptosis and at the same time protects against excessive necrosis when cells are challenged with an agent (menadione) generating reactive oxygen species.

## Experimental Procedures

Reagents used included Fluo-4/Fura-2 (Invitrogen), Tg (Calbiochem), collagenase (Worthington), HEPES and PBS (Lonza), PromoFectin (PromoKine), Bcl-2 pEGFP-C1 (Addgene 17999) and pcDNA3-Bcl-2 (Addgene 8768), rabbit anti-Bcl-2 antibodies (Abcam/Cell Signaling), mouse anti-β-actin antibody (Santa Cruz), Bcl-2 siRNA (sc-29215, Santa Cruz), and scrambled siRNA-A (sc-37007, Santa Cruz). Cell culture reagents were supplied by GIBCO, and all other chemicals were purchased from Sigma (UK). Pancreatic acinar cells were isolated from WT or Bcl-2 KO C57BL6/J mice as described previously [24]. Transgenic mice (B6;129S2-Bcl-2) were obtained from The Jackson Laboratory (stock number 002265). All procedures were approved by local ethical review and covered by UK Home Office licenses. AR42J cells (ECACC, 93100618) were maintained in RPMI 1640 medium (GIBCO) [27]. Ca^2+^ measurements were performed with Fluo-4 or Fura-2 [19, 27], cytosolic YC3.60 Cameleon [20, 21], or D1ER Cameleon [16]. [Ca^2+^]_i_ was calculated (Fura-2) and recovery phases were fitted with an exponential decay function [28]. Immunoblotting and immunofluorescence were performed as described previously [5, 26].
