# Supplementary material for: A Novel Role for Bcl-2 in Regulation of Cellular Calcium Extrusion
Source: Curr Biol. 2012 Jul 10;22(13):1241–6. doi: 10.1016/j.cub.2012.05.002 (PMC3396842; doi:10.1016/j.cub.2012.05.002)

**Current Biology, Volume *22***

# Supplemental Information

# A Novel Role for Bcl-2 in Regulation of Cellular Calcium Extrusion

**Pawel E. Ferdek, Julia V. Gerasimenko, Shuang Peng, Alexei V. Tepikin, Ole H. Petersen, and Oleg V. Gerasimenko**

## Supplemental Figure Legends

**Figure S1. Cytosolic Calcium Extrusion Is Substantially Increased in Bcl-2 KO Cells**

(A) Average traces (mean value and standard error) of cytosolic calcium responses to 10µM ACh and 10µM thapsigargin in Fluo-4-loaded pancreatic acinar cells isolated from WT (blue trace, n=11) and Bcl-2 KO (red trace, n=23) mice.

(B) Comparison of the average area under the traces of normalized Fluo-4 fluorescence responses recorded between 200s and 400s from (A) (p=0.002). Error bars represent standard errors.

(C) Average traces (mean value and standard errors) of cytosolic calcium responses to 10µM thapsigargin in Fura-2-loaded pancreatic acinar cells isolated from WT (blue trace, n=35) and Bcl-2 KO (red traces, n=27) mice presented as changes in 340/380 nm ratio. Initial increase is cytosolic [$Ca^{2+}$] induced by thapsigargin; this is followed by $Ca^{2+}$ extrusion across the plasma membrane. The rate of $Ca^{2+}$ extrusion is much faster in Bcl-2 KO cells than in the WT cells.

(D) The graph shows the rate of decrease in Fura-2 340/380 nm ratio is dependent on the temporal ratio values in WT (blue) and Bcl-2 KO (red) pancreatic acinar cells, which corresponds to cytosolic $Ca^{2+}$ extrusion. -d(Ratio)/dt values were calculated from average traces depicted in (C).

**Figure S2. Cytosolic $Ca^{2+}$ Extrusion in Pancreatic Acinar Cells Is Mainly Dependent on the PMCA.**

(A) Normal (WT) pancreatic acinar cells. Average trace (n=18) showing changes in $[Ca^{2+}]_i$ evoked first by application of thapsigargin in the absence of external $Ca^{2+}$ and thereafter by a 300s period of exposure to an external solution containing 5mM $Ca^{2+}$, followed by its removal. $Na^+$ was present in the external solution throughout the whole experiment.

(B) Normal (WT) pancreatic acinar cells; average trace (n=24). Similar protocol as in (A), but $Na^+$ in external solution was substituted by $NMDG^+$ 200 s before exposure to 5mM $Ca^{2+}$. The absence of $Na^+$ does not affect the rate of $Ca^{2+}$ extrusion.

(C) Pancreatic acinar cell form Bcl-2 KO mouse. Typical trace demonstrating changes in $[Ca^{2+}]_i$ evoked by exposure to an external solution containing 5mM $Ca^{2+}$ in the constant presence of $Na^+$ (standard NaHEPES buffer). The ER was emptied beforehand by application of 2µM thapsigargin.

(D) Pancreatic acinar cell form Bcl-2 KO mouse. Typical trace demonstrating changes in $[Ca^{2+}]_i$ evoked by exposure to an external solution containing 5mM $Ca^{2+}$ in the absence of $Na^+$. The ER was emptied by application of 2µM thapsigargin and thereafter NaHEPES was substituted to NMDG-HEPES. The absence of $Na^+$ does not affect the rate of $Ca^{2+}$ extrusion as compared to (C).

(E) Normal (WT) pancreatic acinar cell. Typical trace demonstrating changes in $[Ca^{2+}]_i$ evoked by exposure to an external solution containing 5 mM $Ca^{2+}$ after empting the ER by application of 2µM thapsigargin. The trace is a control for (F).

(F) Normal (WT) pancreatic acinar cell. Typical trace showing inhibition of cytosolic $Ca^{2+}$ extrusion by 1mM $La^{3+}$ as compared to (E). $[Ca^{2+}]_i$ was increased by exposure to 5mM $Ca^{2+}$ in the external solution after empting the ER with 2µM Tg.

**Figure S3. Overexpression of Bcl-2 in AR42J Cells Decreases PMCA-dependent Cytosolic $Ca^{2+}$ Extrusion across the Plasma Membrane**

(A) Typical trace showing changes in $[Ca^{2+}]_i$ in a control AR42J cell transfected with cytosolic Cameleon D1. The ER store was depleted with 10µM cyclopiasonic acid (CPA) followed by substitution of $Na^+$ in the external solution to $NMDG^+$ (in order to provide inhibition of NCX). Elevated $[Ca^{2+}]_i$ decreased towards the baseline values after removal of 10mM $Ca^{2+}$ from the external solution.

(B) An AR42J cell overexpressing Bcl-2. Similar protocol as in (A). The rate of $Ca^{2+}$ extrusion after removal of 10mM external $Ca^{2+}$ is substantially slower than in the control AR42J cell (shown in (A)).

(C) Typical trace showing changes in $[Ca^{2+}]_i$ in a Fura-2-loaded control AR42J cell. The ER store was depleted with 10µM cyclopiasonic acid (CPA); $Na^+$ was substituted to $NMDG^+$ in the external solution 50s before the cell was briefly exposed to high (10mM) extracellular $Ca^{2+}$. Elevated $[Ca^{2+}]_i$ decreased towards the baseline values after removal of 10mM $Ca^{2+}$ from the external solution.

(D) An AR42J cell overexpressing Bcl-2. Similar protocol as in (C). The rate of $Ca^{2+}$ extrusion after removal of 10mM external $Ca^{2+}$ is much slower than in the control cell (shown in (A)).

(E) The bar chart compares half times ($\tau_{1/2}$) of $[Ca^{2+}]_i$ decrease towards the resting level following removal of external $Ca^{2+}$ in Fura-2-loaded control AR42J cells (blue bar, n=44) and in AR42J cells overexpressing Bcl-2 (purple bar, n=127). Typical traces were presented in (C) and (D).

**Figure S4. Localization of Bcl-2 in Pancreatic Acinar Cells and AR42J Cells.**

(A) Coimmunolocalization of the Bcl-2 and the PMCA in a fixed preparation in a cluster of pancreatic acinar cells. The cells were immunostained with antibodies against Bcl-2 (Aa) and the PMCA (Ab); overlaid in (Bc).

(B) (Ba) A section of a pancreatic acinar cell expressing fusion protein Bcl-2-GFP as shown in green. (Bb) The plasma membrane was stained with FM 1-64, which is shown in red. (Bc) Overlay of green and red fluorescence demonstrates partial colocalization (yellow) at the plasma membrane.

(C) Coimmunolocalization of the Bcl-2-GFP and the PMCA in a fixed preparation of AR42J cells. The cells are expressing fusion protein Bcl-2-GFP – shown in green (Ca); and were immunostained with antibodies against the PMCA (red) (Cb); overlaid in (Cc).

# Supplemental Figure 1

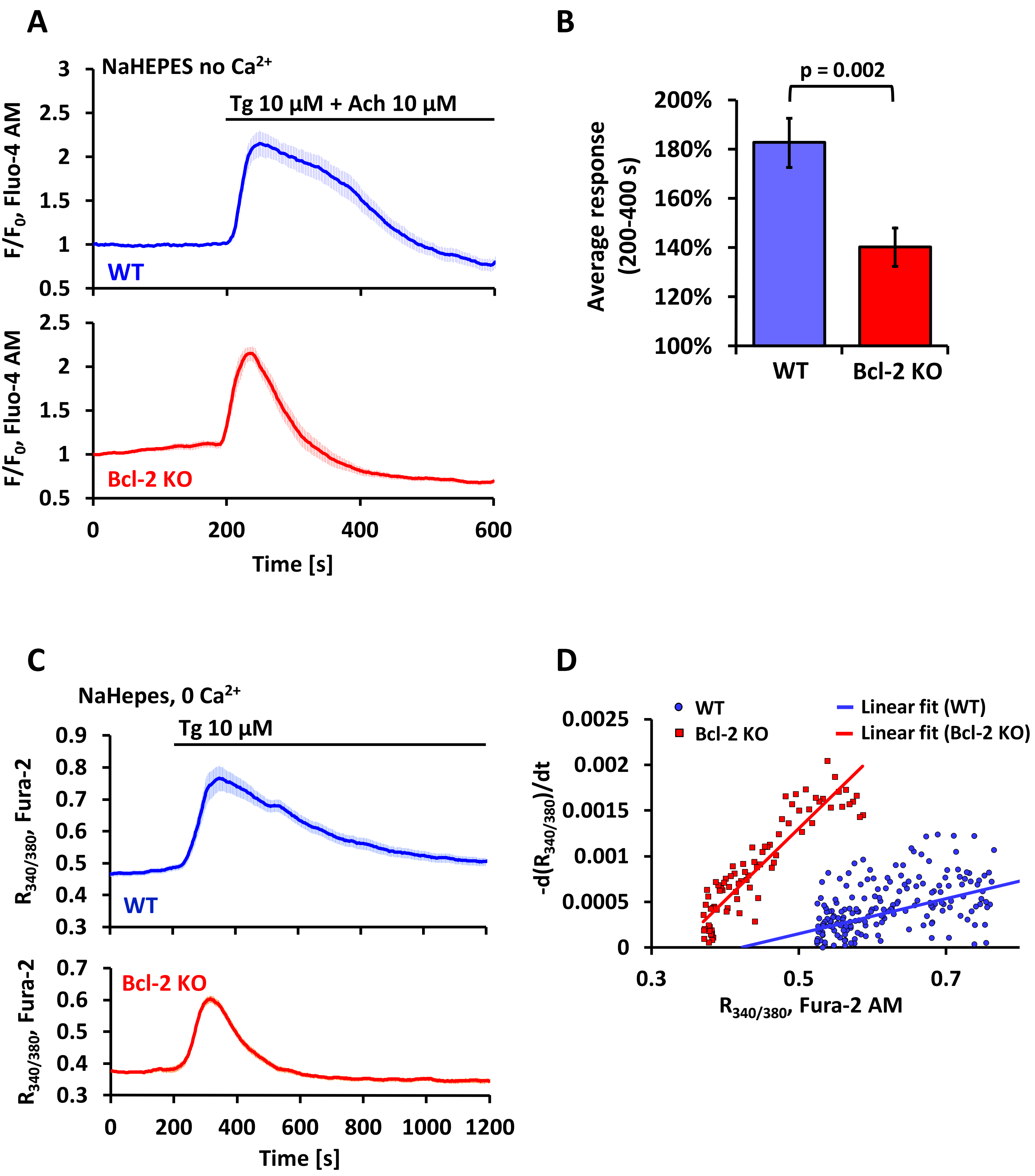

Supplemental Figure 2

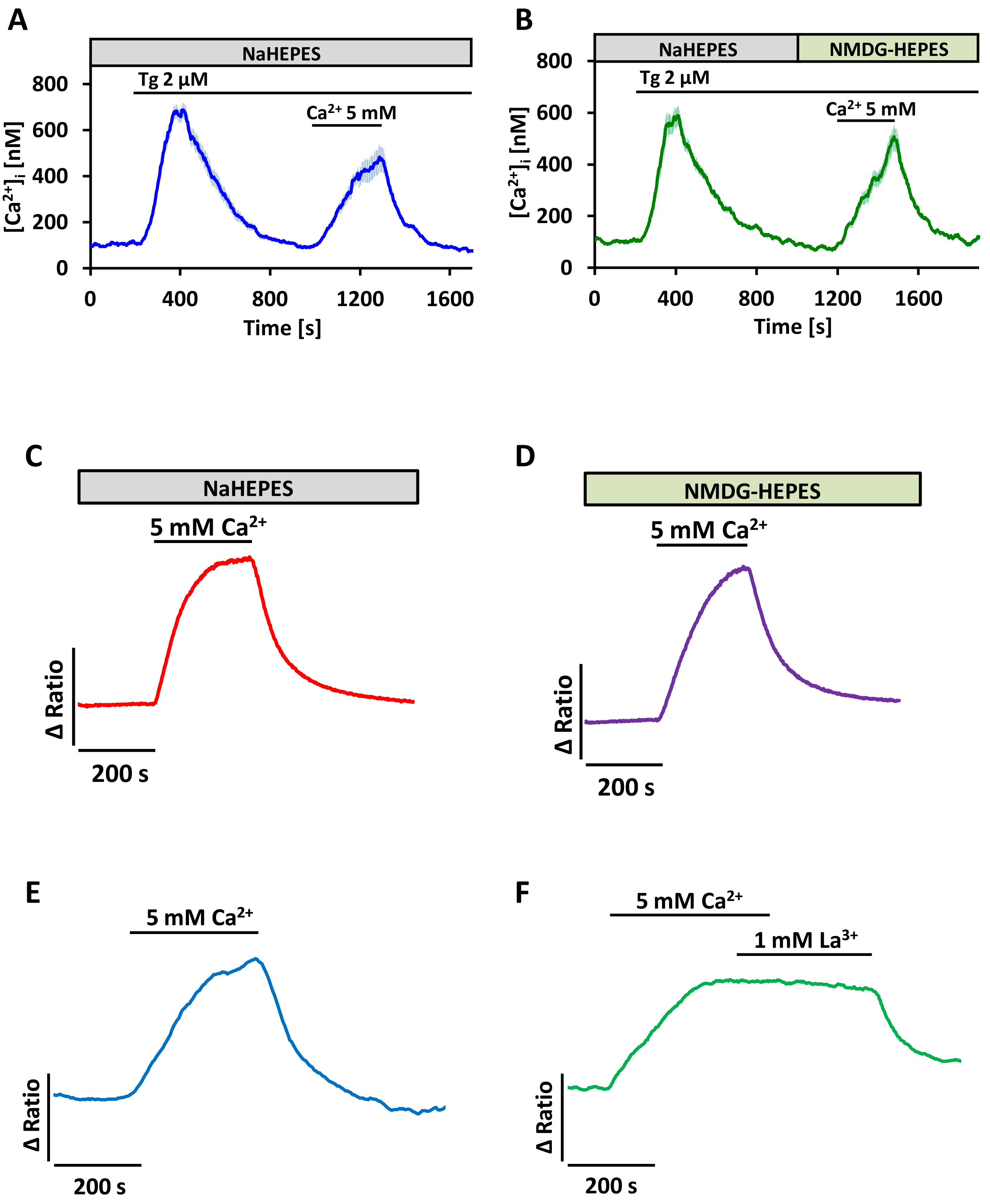

# Supplemental Figure 3

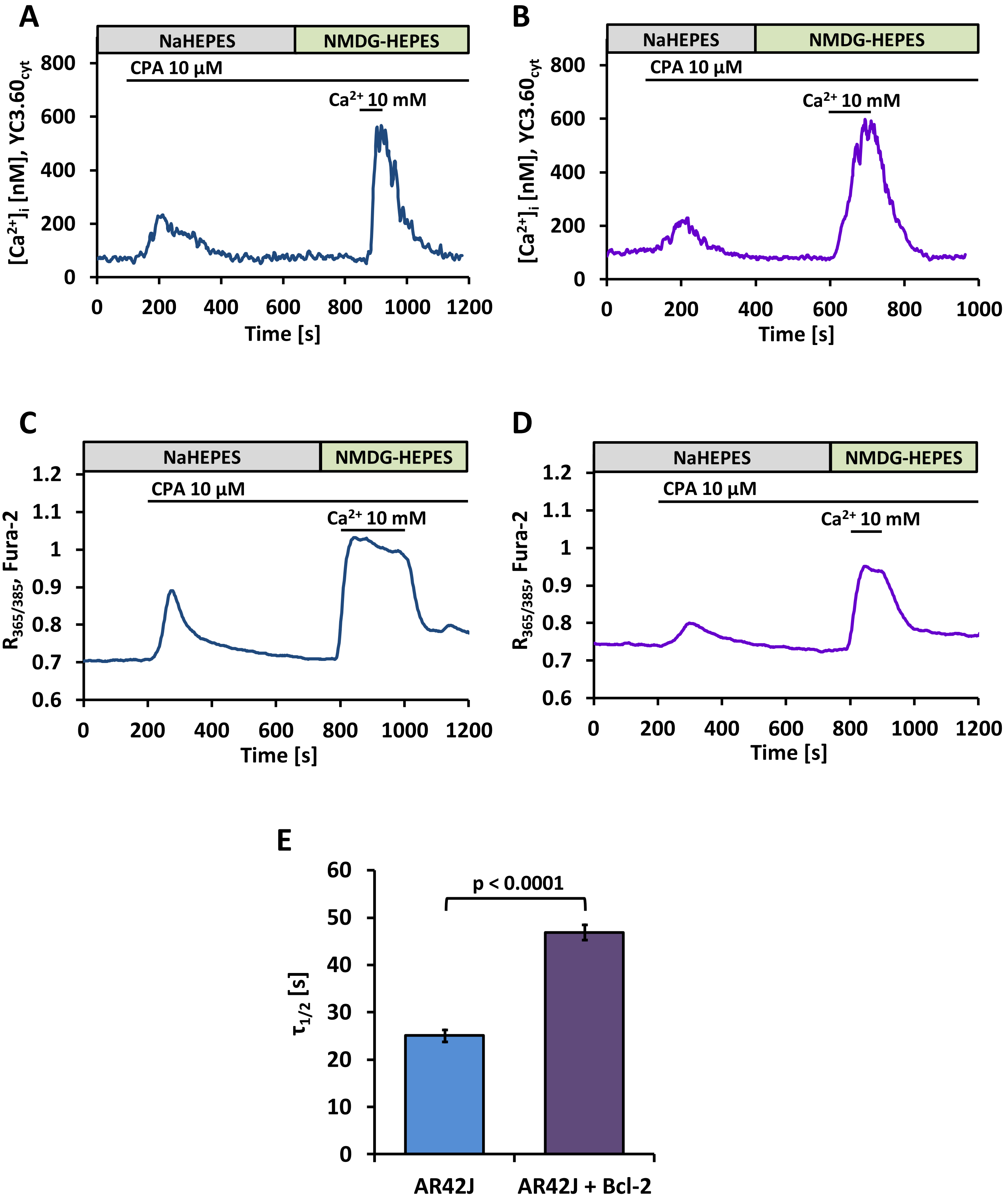

Supplemental Figure 4

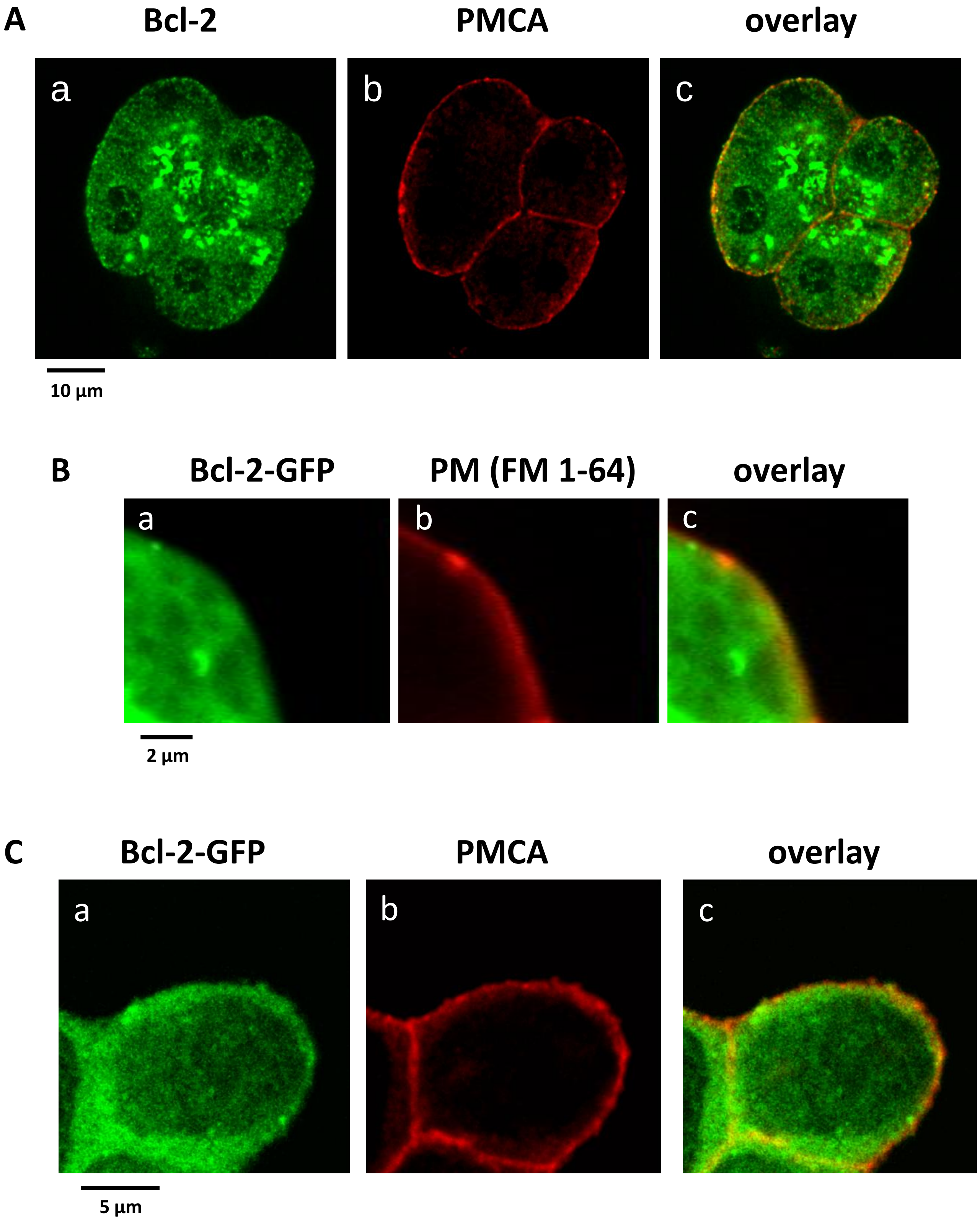

Supplement: Document S1. Figures S1–S4 [file mmc1.pdf]
